# The efficacy of *Nigella sativa* L. oil on serum biomarkers of inflammation and oxidative stress and quality of life in patients with knee osteoarthritis: A parallel triple‐arm double‐blind randomized controlled trial

**DOI:** 10.1002/fsn3.3708

**Published:** 2023-10-07

**Authors:** Afsaneh Amirtaheri Afshar, Vahideh Toopchizadeh, Neda Dolatkhah, Fatemeh Jahanjou, Azizeh Farshbaf‐Khalili

**Affiliations:** ^1^ Faculty of Medicine Tabriz University of Medical Sciences Tabriz Iran; ^2^ Physical Medicine and Rehabilitation Research Center, Aging Research Institute Tabriz University of Medical Sciences Tabriz Iran

**Keywords:** clinical trial, inflammatory biomarkers, *Nigella sativa* L., osteoarthritis, oxidative stress, quality of life

## Abstract

The aim of this double‐blind clinical trial was to investigate the effects of *Nigella sativa* oil on serum inflammatory and oxidative stress biomarkers and quality of life in patients with knee osteoarthritis (OA). Forty‐five patients who met the eligibility criteria were randomly divided into three groups with a ratio of 1:1:1. The first group received 2.5 mL oral *N. sativa* oil twice/day plus placebo topical oil, the second group received 2.5 mL topical *N. sativa* oil three times/day plus placebo oral oil, and the third group received oral and topical oil placebos. There were no intergroup differences in baseline characteristics. After 6 weeks of supplementation, oral *N. sativa* caused a significant improvement in the serum levels of hs‐CRP (*p* = .003), MDA (*p* = .003), and TAC (*p* = .001). Oral *N. sativa* oil compared to placebo (aMD (95% CI): −0.81 (−1.45 to −0.19); *p* = .012) and topical *N. sativa* oil [aMD (95% CI): −0.76 (−1.38 to −0.15); *p* = .016] significantly reduced hs‐CRP serum levels. Significant improvements were observed in the general, physical, and mental health subscales in the oral and topical *N. sativa* oil compared to the placebo group (*p* < .05). The six‐week oral *N. sativa* oil supplementation was effective in improving inflammatory biomarkers in knee OA. Both oral and topical *N. sativa* oil increased the quality of life.

## Graphical abstract

In this randomized controlled trial, oral *N. sativa* oil decreased the serum levels of hs‐CRP and MDA and increased the serum level of TAC. However, this effect was significant compared to the placebo only in hs‐CRP. Both topical and oral *N. sativa* significantly improved the scales and subscales of quality of life compared to placebo. Based on the results, *N. sativa* seems an effective adjuvant therapy for OA without any important side effects.

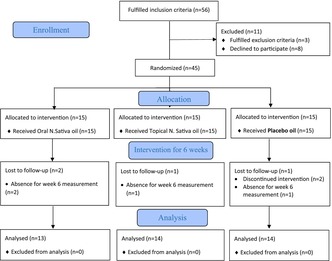

## 1 INTRODUCTION

Osteoarthritis (OA) of the knee is a common degenerative musculoskeletal disease and a cause of disability worldwide, which often affects the elderly. This disease causes pain, physical disability, and reduced quality of life and consumes a significant amount of health system resources and budgets (van Schoor et al., 2005). In the United States, after coronary artery disease, OA is the most common cause of disability and inability to do work in men over 50 years old (Nadji & Akhlaghi, 2012). In recent years, the increase in the elderly population has been relatively associated with the increase in OA patients (Brooks, 2002). It is estimated that 68% of knee OA symptoms are seen in adults over 30 years old (Wood et al., 2013).

Articular cartilage damage accompanied by inflammation, joint swelling, stiffness, pain, loss of mobility, and long‐term disability due to poor innate healing ability is the underlying pathology in OA (Kalamegam et al., 2018; Mobasheri et al., 2014). Conventional treatment comprises modification of lifestyle factors, exercises, cyclooxygenase inhibitors analgesics, especially in early OA with mild cartilage damage (Ondrésik et al., 2017), and surgical management (Quinn et al., 2018).

Today, herbal medicines are used as an alternative treatment with fewer side effects or as a complementary treatment (Aghapour, 2013). The black seed plant with the scientific name *Nigella sativa* belongs to the Ranunculaceae family (Hawsawi et al., 2001).

The phytochemicals in *N. sativa* include a yellowish, volatile oil (0.5%–1.6%), and a fixed oil (35.6%–41.6%) containing unsaturated fatty acids (eicosadienoic, arachidonic, linoleic, and linolenic), stigmasterol, campesterol, α‐spinasterol, β‐sitosterol, limonene, and citronellol (Alshwyeh et al., 2022; Hajhashemi et al., 2004). It contains several amino acids, fibers, minerals, and vitamins including niacin, ascorbic acid, thiamin, pyridoxine, folic acid, and phenolics (Mobasheri et al., 2014; Ramadan, 2007). Phytochemical analyses of *N. sativa* showed the presence of more than hundreds of plant compounds which thymoquinone found as the main ingredient of the oil and the most bioactive compound with extensive therapeutic benefits (Haseena et al., 2015).

Antioxidant, anti‐inflammatory, antihistaminic, and immune system boosting effects have been reported for *N. sativa* oil and extract. Previous studies have reported several pharmacological effects such as reducing blood sugar, fat, and high blood pressure, excreting bile and uric acid, protecting the liver, kidney, heart, and blood vessel tissues, as well as antimicrobial effects for this plant (Tavakkoli et al., 2017). According to these effects, its use in people with OA seems to be effective.

In laboratory mice, oral or injectable use of oil extract of *N. sativa* had analgesic and anti‐inflammatory effects (Hajhashemi et al., 2004). A study investigating the anti‐inflammatory effects of *N. sativa* on rabbits with OA showed that intra‐articular injection of *N. sativa* improved the macroscopic view of the involved joint because of the anti‐inflammatory effects without any specific side effects (Turhan et al., 2019). In a clinical trial, oral administration of *N. sativa* oil was associated with increased levels of the anti‐inflammatory cytokine Interleukin‐10 (IL‐10). However, it had no significant effect on serum Tumor necrosis factor‐alpha (TNF‐α) (Hadi et al., 2016).

Considering the high prevalence of knee OA all over the world and considering the role of inflammation in degenerative diseases associated with aging and the anti‐inflammatory properties of *N. sativa*, this study aimed to determine the effect of oral and topical *N. sativa* oil compared to placebo on serum inflammation (high‐sensitivity C‐reactive protein (hs‐CRP)) as primary outcome, and oxidative stress (total antioxidant capacity (TAC) and malondialdehyde (MDA) biomarkers) and quality of life as secondary outcomes in people with knee OA.

## 2 MATERIALS AND METHODS

### 2.1 Study design and participants

The present study was a double‐blind clinical trial study with three parallel arms in which the participant, the researcher, and the care providers were blinded. This study has been approved by the ethics committee of Tabriz University of Medical Sciences (IR.TBZMED.REC.1395.1291) and has been registered with the code (IRCT20081004001292N5) on the clinical trials website of the Iranian Registry of Clinical Trials. The target population consisted of all patients with mild‐to‐moderate knee OA who were referred to the physical medicine and rehabilitation outpatient clinic in Shohada and Imam Reza Hospital, Tabriz, Iran. The studied participants were selected through convenience sampling, taking into account the eligibility criteria if they were willing to enter the study. The study sampling was conducted from October 2019 to September 2020. Data analysis, interpretation, manuscript writing, and its final draft readiness were completed at the end of 2022.

### 2.2 Eligibility criteria

Men and women over without age limit with mild and moderate knee OA based on the American College of Rheumatology (ACR) criteria (Altman et al., 1986) or Kellgren‐Lawrence radiological criteria (grades 1, 2, and 3) (Kohn et al., 2016) were included in the study. Exclusion criteria were patients with rheumatologic problems such as rheumatoid arthritis, history of surgery on the knee joint, history of fractures in the bones of the lower limbs with involvement of the knee joint surface, severe knee OA (radiology score 4), people with a history of kidney and liver disease according to the patient's statement, people taking anticoagulant drugs, people with neuropathy and sensory disorders and skin disease in the knee area, and people who for any reason were not able to cooperate to complete the questionnaire.

### 2.3 The sample size

Based on Rashidmayvan's study on the effectiveness of *N. sativa* oil on serum level of inflammatory index (Rashidmayvan et al., 2019), taking into account, m1 = 2.09, m2 = 1.65, SD1 = 0.46, SD2 = 0.42, significance level 0.05, power 0.8, 12 people were calculated, and considering 20% for the possibility of dropping, the final sample size was estimated to be 15 people in each group and 45 in total.

### 2.4 Randomization and intervention

The number of 45 patients with knee OA who met the study eligibility criteria was randomly divided into three groups with a ratio of 1:1:1 using a randomization block method with a block size of 6 and 9. The act of randomization and generating the allocation sequence was done by a member of the research team who was outside of any clinical interventions. All patients were given the required information regarding the properties of *N. sativa* and placebo oil, possible side effects, the method and duration of the experiment, and the possibility of the volunteers receiving medication or a placebo, and a written consent form was obtained from them. After obtaining informed consent from the patients, a demographic and baseline information questionnaire was completed for them.

*Nigella sativa* oil was prepared by cold press machine (Barij Essence Pharmaceutical Company, Iran, batch number 402296), and mineral oil was purchased from a pharmacy. 0.1 mL of a mixture containing chlorophyll and red pepper extract in oil‐soluble amounts was added in equal proportion to 150 mL of *N. sativa* oil as an active oil and mineral oil as a placebo to give it a similar appearance and smell. The active and placebo oils were then packaged in a dark‐colored 60 mL glass by a member of the research team who was outside of any clinical intervention. There was no difference in appearance between the oils given to the patients. For each participant, two glasses were considered (one for topical use and one for oral consumption), and these two containers were numbered from 1 to 45 based on the sequence produced by the mentioned person. Individuals received glasses numbered from 1 to 45 in the order of entering the study and were randomly assigned to study groups. Therefore, allocation concealment and blinding were done in this study.

The participants of the first group underwent supplementation with oral *N. sativa* oil at a dose of 2.5 mL twice a day (Fallah Huseini et al., 2013) and placebo topical oil for 6 weeks. The second group used *N. sativa* oil topically and placebo edible oil twice a day, and the third group or control received 2.5 mL of placebo edible oil and placebo topical oil. In the topical use of the active oil and placebo, the patients were asked to apply a sufficient amount of the oil on the skin of the knee area and around it three times a day for 6 weeks, and not remove it for 25 min. During the study, all patients benefited from the usual medical and physical treatments, including vitamin D and calcium, strengthening the quadriceps muscle, and advice to correct the destructive habits of OA.

### 2.5 The measurement of variables

Demographic baseline information of the participants: It was collected using a general characteristic questionnaire through a face‐to‐face interview. Individual information included age, sex, side of knee involvement, consumption of different supplements, and taking a special diet. At the beginning of the study, people's height was measured using a Seca wall height meter with an accuracy of 1 mm while standing next to the wall without shoes and the shoulders in normal conditions. Weight was measured using a Seca digital floor scale with an accuracy of 100 g and a capacity of 220 with minimal clothing. Then, the body mass index of people was calculated using the formula of weight in kilograms divided by the square of height in meters. A daily diary was used to record the use of oral and topical supplements, as well as a self‐report checklist for recording drug side effects.

Twenty four hour food recall questionnaire: In order to evaluate the diet, the food received by the participants in 3 days (two working days and one weekend day) at the beginning of the study was determined using the 24‐h food recall questionnaire through a face‐to‐face interview. The average of three questionnaires is considered and analyzed. Food units were converted to grams per day using the book “Home Scales Guide.” Also, each food and drink was coded and entered into Nutritionist IV nutritional software (First Databank, San Bruno, CA) modified for Iranian foods to evaluate the amount of energy and nutrients received by the participants.

The Short Form 36 Health Survey Questionnaire (SF‐36): The SF‐36 Short Form Questionnaire is a health measurement tool that can measure the quality of life (QoL) well (Brazier et al., 1992). We used SF‐36 to indicate the QoL of study participants before and immediately after the intervention. The SF‐36 is a 36‐item scale that measures eight domains of health status: physical Function (PF), role limitations due to physical problems (RP), bodily pain (pain), general health (GH), vitality, social functioning (SH), role limitations due to emotional problems (RE), and emotional well‐being (EW). The total score in each subscale of SF‐36 ranges from 0 to 100. The two subscales of physical health (PH) and mental health (MH) are obtained from a sum of the above first and second four subscales. Higher scores indicate better quality in each subscale (Bunevicius, 2017).

### 2.6 The measurement of biochemical parameters

Fasting blood samples (5 mL) were taken before and after the intervention in a state of fasting for 8–10 h at 8–10 am and were centrifuged at 3500 rpm for 10 min. The samples were kept in a freezer at −70°C until the measurements. To reduce the error, each biochemical parameter was measured by one person and a specific device. Serum levels of hs‐CRP were determined using commercially available cytokine ELISA kits (DIASource, Belgium) following the instructions of the manufacturers at 450 nM wavelength in an ELISA plate reader apparatus (Awareness, Statfax‐2100 model, USA) (Hadi et al., 2016). The Measurement of serum TAC was carried out by colorimetric/photometric method and using a Navand Salamat kit (Rice‐Evans & Miller, 1994). Serum MDA concentration was determined using the thiobarbituric acid method described by Bilici (Del Rio et al., 2003).

### 2.7 Statistical analysis

The normality of data distribution was investigated by the Kolmogorov–Smirnov test. Descriptive statistics were used to compare the basic characteristics of the participants in the three studied groups. To compare baseline values, one‐way analysis of variance (ANOVA) was used for data with normal distribution, the Kruskal–Wallis test for quantitative data with non‐normal distribution, and the Chi‐square test for nominal qualitative data. Paired sample *t*‐test was used for intra‐group comparisons, and univariate analysis of covariance (ANCOVA) was used for intergroup comparisons adjusted for baseline values. The significant level was considered as *p* < .05. The statistical software IBM SPSS Statistics version 17 was used for the statistical analysis of the data. All analyses were done based on an intention‐to‐treat approach.

## 3 RESULTS

**FIGURE 1:**
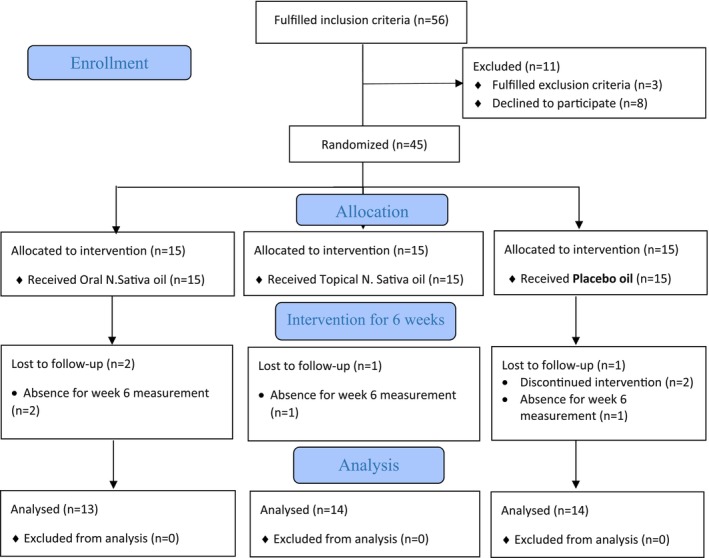
Study flow diagram.

A total of 56 people were initially evaluated to participate in the study. Among these, 45 people were eligible to participate in the study and randomly assigned to three study groups: oral *N. sativa*, topical *N. sativa*, and placebo. Two people in the oral nigella group, one person in the topical nigella group, and one person in the placebo group were unable to complete the study due to unwillingness to continue (Figure 1).

### 3.1 Baseline characteristics

The average age of the participants was 53.76 ± 8.10 years in the oral *N. sativa* group, 53.87 ± 6.59 years in the topical *N. sativa* group, and 54.94 ± 10.49 years in the control group (placebo). There was no statistically significant difference between the study groups in terms of age, sex, body mass index, nutritional supplement consumption, special diet, dietary macronutrients, and dietary micronutrients (*p* < 0.05) (Table 1).

**TABLE 1:** Baseline characteristics of the participants among study groups.

Variable	Oral *N. sativa* (*n* = 15) Mean ± SD	Topical *N. sativa* (*n* = 15) Mean ± SD	Placebo (*n* = 15) Mean ± SD	*p*‐Value
Age (year)	53.76 ± 8.10	53.87 ± 6.59	54.94 ± 10.49	.912^a^
Sex				
Male	2 (13.3%)	1 (6.7%)	2 (13.3%)	.599^b^
Female	13 (86.7%)	14 (93.3%)	13 (86.7%)	
Osteoarthritis				
Right knee	2 (11.8%)	1 (6.7%)	3 (18.8%)	.640^b^
Left knee	1 (5.9%)	2 (13.3%)	3 (18.8%)	
Bilateral	14 (82.4%)	12 (80.0%)	10 (62.5%)	
Weight (kg)	81.50 ± 18.13	78.18 ± 10.83	81.86 ± 10.43	.528^a^
Height (m)	161.12 ± 5.89	159.47 ± 6.64	162.81 ± 8.53	.429^a^
BMI (kg/m^2^)	31.85 ± 4.70	30.70 ± 4.35	30.58 ± 5.53	.714^a^
Dietary supplement intake	9 (60.0%)	8 (53.3%)	10 (66.7%)	.876^c^
Supplement type				
Vitamin D	2 (13.3%)	3 (20.0%)	2 (13.3%)	.585^b^
Calcium‐Vit D	5 (33.3%)	3 (20.0%)	5 (33.3%)	
Glucosamine	1 (6.6%)	0 (0.0%)	1 (6.6%)	
Vitamin B	0 (0.0%)	1 (6.6%)	0 (0.0%)	
Calcium‐Vit B1	0 (0.0%)	1 (6.6%)	2 (13.3%)	
Vitamin D‐Rabonex	1 (6.6%)	0 (0.0%)	0 (0.0%)	
Taking special diet	0 (0.0%)	1 (6.6%)	0 (0.0%)	.313^b^
Dietary intake				
Carbohydrates (g/day)	231.68 ± 61.17	219.59 ± 56.20	237.10 ± 65.00	.719^a^
Protein (g/day)	84.51 ± 52.17	72.79 ± 54.12	68.26 ± 39.53	.748^a^
Fat (g/day)	18.73 ± 4.78	17.03 ± 4.75	17.98 ± 5.68	.619^a^
SFAs (g/day)	18.73 ± 4.78	17.03 ± 4.75	17.98 ± 5.68	.644^a^
MUFAs (g/day)	36.29 ± 6.45	33.64 ± 6.73	34.31 ± 8.06	.548^a^
PUFAs (g/day)	25.37 ± 6.02	24.68 ± 5.57	25.33 ± 6.14	.937^a^
Cholesterol (mg/day)	170.51 ± 21.20	165.91 ± 17.80	165.83 ± 25.40	.780^a^
Fiber (g/day)	16.97 ± 6.16	15.02 ± 5.47	16.38 ± 6.48	.654^a^

*Note*: Quantitative variables are reported with mean ± standard deviation and qualitative variables with number (%).

Abbreviations: MUFAs, monounsaturated fatty acids; PUFAs, polyunsaturated fatty acids; SFAs, saturated fatty acids.

^a^ One‐way analysis of variance.

^b^ Fishers' exact test.

^c^ Chi‐square.

### 3.2 The mean serum levels of hs‐CRP, TAC, and MDA

The mean serum levels of hs‐CRP among oral *N. sativa*, topical *N. sativa*, and placebo groups were 4.30 ± 1.26, 4.13 ± 1.25, and 3.56 ± 1.29 at baseline which decreased to 3.30 ± 1.11, 3.92 ± 1.61, and 3.49 ± 1.34 after intervention, respectively. The TAC serum levels in the aforementioned groups were 1.98 ± 0.94, 1.93 ± 1.10, and 2.66 ± 1.73 at baseline that following intervention increased to 3.28 ± 1.24, 2.47 ± 1.17, and 2.94 ± 1.62, respectively. Besides, the pre‐treatment serum levels of MDA among oral *N. sativa*, topical *N. sativa*, and placebo groups were 3.16 ± 1.13, 2.80 ± 1.09, and 2.30 ± 1.06 which reduced to 2.22 ± 0.95, 2.48 ± 1.05, and 2.23 ± 1.10 post‐treatment, respectively.

The consumption of oral *N. sativa* caused a significant decrease in the serum levels of hs‐CRP (*p* = .003) and MDA (*p* = .003), and a significant increase in TAC (*p* = .001). However, the changes in these biomarkers in the other groups were not significant (*p* > .05). The between‐group comparison of data indicated that the use of oral *N. sativa* compared to both placebo (adjusted mean difference (95% confidence interval) (aMD (95% CI): −0.81 (−1.45 to −0.19); *p* = .012)) and topical *N. sativa* oil [aMD (95% CI): −0.76 (−1.38 to −0.15); *p* = .016] groups significantly reduced hs‐CRP serum levels. There was no significant difference between groups in terms of MDA and TAC serum levels after the intervention (*p* < .05) (Table 2).

**TABLE 2:** Serum inflammatory and oxidative stress biomarkers among study groups before and after intervention.

Variable	Oral *N. sativa* (*n* = 13) Mean ± SD	Topical *N. sativa* (*n* = 14) Mean (SD)	Placebo (*n* = 14) Mean (SD)	Adjusted MD (95% CI) Oral N.S/placebo	*p*	Adjusted MD (95% CI) Topical N.S/placebo	*p*	Adjusted MD (95% CI) Oral/topical N.S	*p*
hs‐CRP									
Baseline	4.30 ± 1.26	4.13 ± 1.25	3.56 ± 1.29	–	.227^b^	–	.427^b^	–	.928^b^
End	3.30 ± 1.11	3.92 ± 1.61	3.49 ± 1.34	−0.81 (−1.45 to −0.19)	**.012** ^c^	−0.05 (−0.68 to 0.59)	.881^c^	−0.76 (−1.38 to −0.15)	**.016** ^c^
*p* ^a^	**.003**	.318	.559	–	–	–	–	–	–
TAC									
Baseline	1.98 ± 0.94	1.93 ± 1.10	2.66 ± 1.73	–	.295^b^	–	.274^b^	–	.995^b^
End	3.28 ± 1.24	2.47 ± 1.17	2.94 ± 1.62	0.77 (−0.02 to 1.57)	.056^c^	−0.01 (−0.83 to 0.83)	.982^c^	0.78 (−0.01 to 1.57)	.052^c^
*p* ^a^	**.001**	.134	.279	–	–	–	–	–	–
MDA									
Baseline	3.16 ± 1.13	2.80 ± 1.09	2.30 ± 1.06	–	.073^b^	–	.416^b^	–	.630^b^
End	2.22 ± 0.95	2.48 ± 1.05	2.23 ± 1.10	−0.53 (−1.13 to 0.08)	.085^c^	−0.05 (−0.65 to 0.55)	.864^c^	−0.48 (−1.06 to 0.11)	.109^c^
*p* ^a^	**.003**	.267	.563	–	–	–	–	–	–

*Note*: Bold indicates significance level (*p* < 0.05).

^a^ Paired sample *t*‐test.

^b^ One‐way ANOVA with Tukey's post hoc test.

^c^ ANCOVA adjusted for baseline values.

### 3.3 The quality of life among study groups during the study

The score of PH‐QoL was 173.88 ± 65.42, 190.00 ± 61.33, and 217.72 ± 69.6 in the oral *N. sativa*, topical *N. sativa*, and placebo groups at baseline that improved to 266.68 ± 81.30, 291.43 ± 52.16, and 220.59 ± 72.6 after intervention, respectively. The pre‐intervention score of MH‐QoL was 267.34 ± 90.54, 285.54 ± 66.55, and 301.33 ± 87.5 which changed to 312.04 ± 93.92, 318.63 ± 77.08, and 297.88 ± 89.5 following intervention, respectively. The pre‐ and post‐intervention scores of the rest subscales are indicated in Table 3.

**TABLE 3:** Quality of life among study groups before and after intervention.

Variable	Oral *N. sativa* (*n* = 13) Mean (SD)	Topical *N. sativa* (*n* = 14) Mean (SD)	Placebo (*n* = 14) Mean (SD)	Adjusted MD (95% CI) Oral N.S/placebo	*p*	Adjusted MD (95% CI) Topical N.S/placebo	*p*	Adjusted MD (95% CI) Oral/topical N.S	*p*
PF (0–100)									
Baseline	32.94 ± 18.29	44.66 ± 19.59	50.68 ± 28.51	–	.094^a^	–	.495^a^	–	.076^a^
End	56.76 ± 19.44	64.66 ± 19.59	52.56 ± 24.28	17.2 (7.2 to 27.2)	**.001** ^c^	16.5 (6.7 to 26.3)	**.001** ^c^	0.70 (−9.1 to 10.5)	.887^c^
RP (0–100)									
Baseline	23.52 ± 31.21	26.66 ± 25.81	21.87 ± 30.10	–	.86^b^	–	.892^b^	–	.951^b^
End	63.97 ± 32.44	83.92 ± 18.62	20.31 ± 27.71	–	**.001** ^a^	–	**<.001** ^a^	–	.084^a^
RE (0–100)									
Baseline	72.54 ± 41.22	91.11 ± 26.62	77.08 ± 39.84	–	.933^b^	–	.543^b^	–	.337^b^
End	86.27 ± 33.45	93.33 ± 25.81	75.00 ± 44.72	13.6 (−7.9 to 35.1)	.209^c^	11.1 (−11.3 to 33.5)	.323^c^	2.5 (−19.8 to 24.8)	.824^c^
EF (0–100)									
Baseline	60.14 ± 22.22	60.66 ± 22.35	71.56 ± 15.13	–	.246^b^	–	.300^b^	–	.997^b^
End	73.08 ± 24.23	71.33 ± 24.89	72.18 ± 15.70	11.8 (4.1 to 19.5)	**.003** ^c^	9.6 (1.6 to 17.5)	**.019** ^c^	2.2 (−5.4 to 9.9)	.554^c^
EW (0–100)									
Baseline	62.58 ± 19.99	60.26 ± 21.98	73.00 ± 13.74	–	.262^b^	–	.156^b^	–	.936^b^
End	71.05 ± 21.61	64.80 ± 22.07	71.00 ± 15.49	10.2 (4.6 to 15.8)	**.001** ^c^	6.2 (0.36 to 12.1)	**.038** ^c^	3.4 (−1.6 to 9.6)	.155^c^
SF (0–100)									
Baseline	72.05 ± 19.53	73.50 ± 16.16	79.68 ± 24.52	–	.536^b^	–	.679^b^	–	.978^b^
End	81.61 ± 23.01	89.16 ± 14.84	79.68 ± 22.30	8.1 (−0.7 to 16.9)	.070^c^	14.5 (5.5 to 23.5)	**.002** ^c^	−6.4 (−15.2 to 2.4)	.152^c^
Pain (0–100)									
Baseline	65.29 ± 20.78	66.66 ± 18.36	74.84 ± 16.86	–	.078^a^	–	.060^a^	–	1.000^a^
End	79.70 ± 23.26	81.50 ± 16.63	82.96 ± 17.20	9.1 (0.7 to 17.4)	**.034** ^c^	8.0 (−0.4 to 16.4)	.062^c^	1.1 (−7.0 to 9.1)	.792^c^
GH (0–100)									
Baseline	52.11 ± 16.31	50.00 ± 16.36	65.31 ± 19.36	–	**.023** ^a^	–	**.011** ^a^	–	.797^a^
End	66.23 ± 21.51	62.33 ± 19.26	64.75 ± 21.96	14.7 (5.9 to 23.4)	**.002** ^c^	12.9 (3.7 to 22.1**)**	**.007** ^c^	1.8 (−6.7 to 10.3)	.674^c^
PH (0–400)									
Baseline	173.88 ± 65.42	190.00 ± 61.33	217.72 ± 69.6	–	.145^b^	–	.474^b^	–	.769^b^
End	266.68 ± 81.30	291.43 ± 52.16	220.59 ± 72.6	79.4 (42.8 to 116.0)	**<.001** ^c^	92.1 (54.5 to 129.6)	**<.001** ^c^	−12.6 (−49.2 to 24.0)	.491^c^
MH (0–400)									
Baseline	267.34 ± 90.54	285.54 ± 66.55	301.33 ± 87.5	–	.472^b^	–	.857^b^	–	.809^b^
End	312.04 ± 93.92	318.63 ± 77.08	297.88 ± 89.5	43.1 (5.8 to 80.5)	**.024** ^c^	34.2 (−3.8 to 72.3)	.077^c^	8.9 (−28.6 to 46.5)	.634^c^

*Note*: Higher scores indicate better quality. Physical health is obtained from the sum of the subscales of PF, pain, RP, and GH, and the Mental health is obtained from the sum of the subscales of EW, EF, RE, and SF. Bold indicates significance level (*p* < 0.05).

Abbreviations: EF, emotional function; EW, emotional well‐being; GH, general health; MH, mental health; PF, physical function; PH, physical health; RE, role emotional; RP, role limitations due to physical problems; SF, social functioning.

^a^ Mann–Whitney U.

^b^ One‐way ANOVA with Tukey's post hoc test.

^c^ ANCOVA adjusted for baseline values.

**FIGURE 2:**
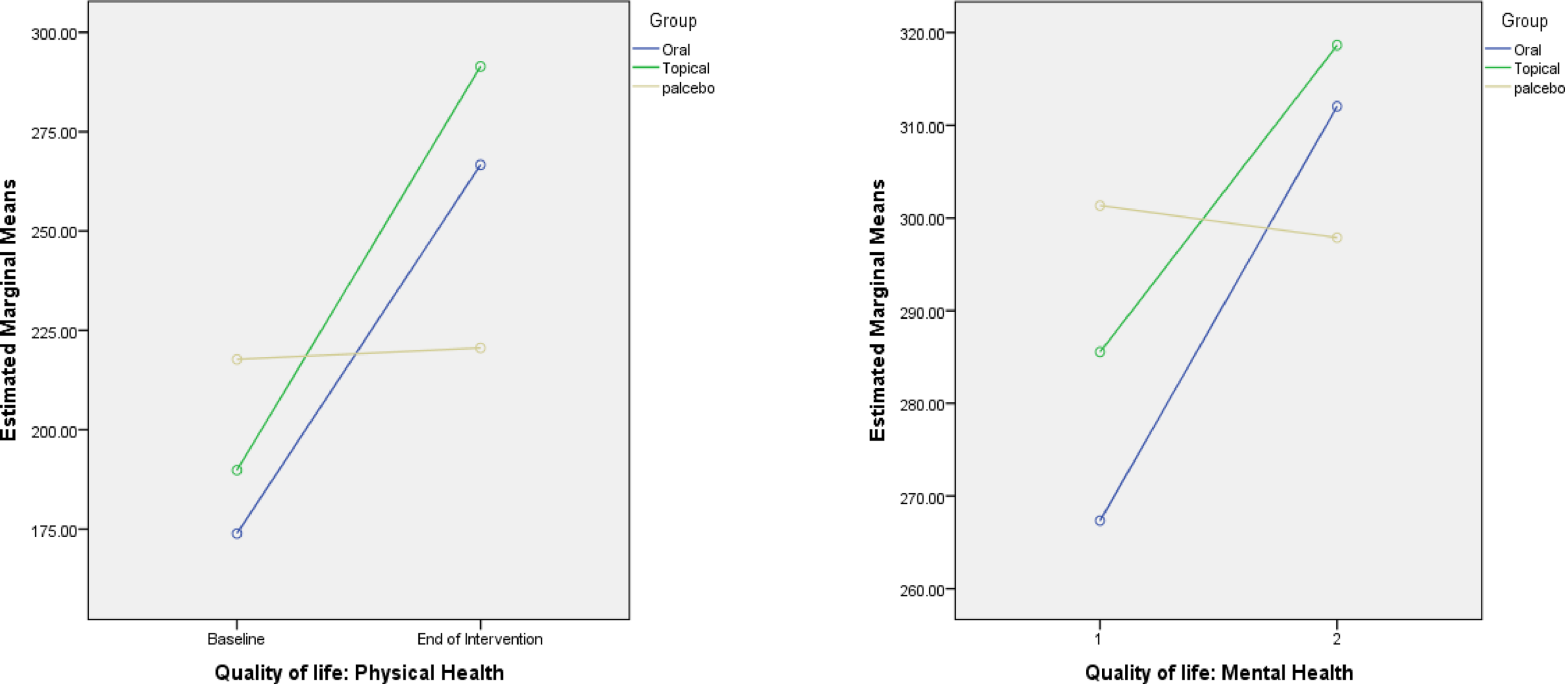
The changes of quality of life score in physical and mental health subscale during 6 weeks of intervention with *Nigella sativa* by study groups.

The QoL scores had statistically significant increases in the oral *N. sativa* compared to the placebo group regarding PF (*p* = .001), RP (*p* = .001), EF (*p* = .003), EW (*p* = .001), pain (*p* = .034), and GH (*p* = .002) at the end of the intervention. Also, the post‐intervention QoL scores regarding the PF (*p* = .001), RP (*p* < .001), EF (*p* = .019), EW (*p* = .038), SF (*p* = .002), and GH (*p* = .007) subscales significantly increased in the topical *N. sativa* compared to the placebo group. There was no significant difference in any of these subscales between the oral and topical *N. sativa* groups after the intervention (*p* > .05). In terms of the overall PH subscale, after the intervention, there was a significant increase in the oral *N. sativa* group [aMD (95% CI): 79.4 (42.8 to 116.0); *p* < .001] and also in the topical *N. sativa* group [aMD (95% CI): 92.1 (54.5 to 129.6); *p* < .001] in comparison with placebo, but in terms of the overall MH subscale, this increase was observed only in the oral *N. sativa* group [aMD (95% CI): 43.1 (5.8 to 80.5); *p* = .024] (Table 3 and Figure 2).

### 3.4 The reported side effects and medication adherence

Regarding the side effects, in the oral *N. sativa* oil group two cases of eczema, and in the topical *N. sativa* group, one case of contact dermatitis was reported. All of these side effects were mild and mainly happened at the beginning of use.

The medication adherence rate based on the medication checklist was 86% in the oral *N. sativa* oil group, 88% in the topical *N. sativa* oil group, and 84% in the placebo group.

## 4 DISCUSSION

This study was conducted to investigate and compare the effect of oral and topical *N. sativa* oil in improving some inflammatory and oxidative stress indicators as well as the quality of life of people with knee OA.

In this study, we hypothesized that the oral and topical using of *N. sativa* oil compared to placebo ameliorates the serum inflammatory biomarker (hs‐CRP) as the primary outcome as well as improves the serum TAC and MDA, and quality of life as secondary outcomes in people with knee OA. According to the results, the consumption of oral *N. sativa* oil caused a significant decrease in the serum levels of hs‐CRP and MDA and a significant increase in TAC. However, the use of oral *N. sativa* oil compared to placebo and topical *N. sativa* oil significantly reduced the serum levels of only hs‐CRP. Regarding the quality of life, although the majority of subscales and the overall PH at the end of the intervention increased in the oral and topical *N. sativa* oil group compared to the placebo, in terms of the overall MH, this increase was observed only in the oral *N. sativa* oil group. Therefore, our hypothesis regarding the efficacy of oral use of *N. sativa* oil on inflammatory biomarkers and quality of life is confirmed. In the case of topical use, it is not confirmed for inflammatory and oxidative stress biomarkers, but it is approved for quality of life in most areas.

Chronic inflammation is a prevalent underlying factor in most age‐related degenerative diseases like OA. Since conventional treatments only partially relieve symptoms, herbal supplements are being widely investigated (Kalamegam et al., 2020). The *N. sativa* oil has a large variety of constituents including thymoquinone, which is its main and not sole bioactive component (Fatima Shad et al., 2021).

The potential anti‐inflammatory mechanism of TQ may be attributed to the suppression of the oxidative byproducts of arachidonic acid synthesis, namely thromboxane B2 and leukotriene, through the inhibition of both cyclooxygenase and lipoxygenase enzymes (Mansour & Tornhamre, 2004). Moreover, the alcoholic extract and essential oil derived from N. sativa have exhibited a significant painkiller effect. Additionally, the potent antioxidant characteristic of this seed has gained remarkable attention in regard to its possible function as a dietary supplement with negligible adverse outcomes (Yimer et al., 2019). *N. sativa* and its active constituent, thymoquinone, cause a reduction in oxidative stress through blocking calcium channel and increasing urine output (Leong et al., 2013).

In line with our study, the results of an in vitro study revealed that TQ, an active ingredient of Nigella sativa, has moderate anti‐inflammatory effects in OA and therefore has the potential to be used in therapeutics. According to their results, TQ either alone or in combination with conventional pharmacological agents will help in reducing the inflammation associated with inflammatory joints and age‐related degenerative diseases (Kalamegam et al., 2020).

Studies examining the effect of topical application of *N. sativa* oil on improving symptoms of hip or knee OA are rare. Systemic and local administration of black seed oil has shown analgesic and anti‐inflammatory effects in mice (Hajhashemi et al., 2004).

In an experimental study conducted on rabbits, in one group *N. sativa* and in another group saline were injected intra‐articularly after surgery, and the effects of these two interventions on the joint were examined macroscopically and microscopically. The results of this study indicated the macroscopic improvement of the involved joint and the anti‐inflammatory effects of *N. sativa* on that joint, which was consistent with the results of our study in case of oral use of *N. sativa*. Also, no special complication was observed after *N. sativa* injection (Turhan et al., 2019).

Hadi et al. studied the anti‐inflammatory and antioxidant effects of *N. sativa* oil in rheumatoid arthritis patients (Hadi et al., 2016). Consistent with the results of the present study, the oral administration of *N. sativa* oil at the rate of 1 gram per day in the form of capsules was associated with an increase in the level of the anti‐inflammatory cytokine IL‐10. However, it had no significant effect on TNF‐α serum level. Also, in the study of Rashidmayvan et al., the administration of *N. sativa* oil compared to paraffin as a placebo for 8 weeks in 44 patients with alcoholic fatty liver led to a significant decrease in hs‐CRP, TNF‐α, and IL‐6 (Rashidmayvan et al., 2019). It has also been shown that administration of 3 grams of black seed oil per day along with a low‐calorie diet for 8 weeks in 90 obese women aged 25–50 years significantly reduced serum levels of TNF‐α and hs‐CRP compared to placebo. However, no significant change in IL‐6 serum level was observed (Mahdavi et al., 2016). Although the results of these three human studies were in line with our results, the type of disease was different from our research. In our study, the daily dose of *N. sativa* oil was higher, but the duration of the intervention was shorter.

In contrast to the results of the present study, in the study of Nikkhah‐Bodaghi et al., the administration of 2 g of *N. sativa* powder per day in 46 patients with ulcerative colitis for 6 weeks did not improve the serum levels of inflammatory and oxidative markers TNF‐α, hs‐CRP, and serum TAC, but it decreased the serum level of MDA (Nikkhah‐Bodaghi et al., 2019). This difference can be attributed to the difference in the daily dose received and the type of disease and the severity of inflammation in the participants. Also, we used the oil of *N. sativa* in our study, but its powder was used in the mentioned study. However, the duration of the intervention was similar in both studies.

Based on the results, the changes in scores on most scales and subscales of the quality of life were significantly different between the three study groups. In most cases, the improvement in the oral and topical *N. sativa* group was significantly more than placebo. Both oral and topical interventions were equally successful in improving physical and mental health subscales, and this improvement was more evident in the overall psychological scale of MH in the oral group. The progressive nature of OA and the resulting pain and disability significantly affect the ability of the affected person to perform daily activities (Nguyen et al., 2011). These conditions cause a decrease in activity and mobility in the patient, which inevitably decreases the level of his/her quality of life (Salaffi et al., 2005). There is growing evidence that mental health and quality of life disorders in patients with OA occur due to dysregulation of the hypothalamic–pituitary–adrenal axis and inflammatory cytokines such as interleukin‐6 and TNF‐α (Eller‐Smith et al., 2018). Chronic inflammatory cytokines increase is associated with an increased risk of OA, sleep disorders, depression, and reduced quality of life.

According to our knowledge, no study has been published that examines the effect of *N. sativa* oil on the quality of life in patients with OA. However, these effects have recently been investigated in other disorders and conditions such as ulcerative colitis (Nikkhah‐Bodaghi et al., 2019), premenstrual syndrome (Maskani et al., 2020), and gastritis caused by Helicobacter pylori (Alizadeh‐Naini et al., 2020) and have brought promising results.

Based on the authors' search in reliable international databases until the time of writing and submitting this manuscript, the present study is the first study investigating the effect of oral and topical *N. sativa* oil compared to placebo on the quality of life of patients with knee OA and can be a basis for future studies to determine the exact dose–response of *N. sativa* oil.

The use of unit dosage of *N. sativa* oil makes it difficult to determine its most effective dose for clinical use or inclusion in the diet. The majority of people and patients examined were female, which can make it difficult to generalize the results to all patients with OA. The duration of the study was relatively short, and the sample size was small. Therefore, it is recommended to conduct studies with follow‐up intervention and a larger number of samples in the future.

## 5 CONCLUSIONS

Oral *N. sativa* decreased the serum levels of hs‐CRP and MDA and increased the serum level of TAC. However, this effect was significant compared to the placebo only in hs‐CRP. Topical and oral *N. sativa* both significantly improved the scales and subscales of quality of life compared to placebo, except for the subscale RE in both groups and overall MH for the topical group. Based on the results, *N. sativa* seems an effective adjuvant therapy for OA without any important side effects. Conducting future studies with a larger sample size is recommended.

## AUTHOR CONTRIBUTIONS

**Afsaneh Amirtaheri Afshar:** Conceptualization (equal); data curation (equal); formal analysis (equal); investigation (equal); writing – original draft (equal). **Vahideh Toopchizadeh:** Conceptualization (equal); data curation (equal); funding acquisition (equal); project administration (equal); supervision (equal); writing – review and editing (equal). **Fatemeh Jahanjou:** Data curation (equal); formal analysis (equal); writing – review and editing (equal).

## FUNDING INFORMATION

This study has been funded by the Physical Medicine and Rehabilitation Research Center of Tabriz University of Medical Sciences, Iran (grant ID: 58047). The funding body had no role in the design of the study and collection, analysis, and interpretation of data and in writing the manuscript.

## CONFLICT OF INTEREST STATEMENT

The authors declare that they have no competing interests.

## ETHICS STATEMENT

This study has been approved by the ethics committee of Tabriz University of Medical Sciences (IR.TBZMED.REC.1395.1291). All methods were performed in accordance with the relevant guidelines and regulations (Declaration of Helsinki).

## CONSENT TO PARTICIPATE

An informed consent form was signed by all participants before recruiting for the study.

## TRIAL REGISTRATION

This study was registered under the number IRCT20081004001292N5 on the Iranian Registry of Clinical Trials website. Date of first registration: 22/01/2019.

## Data Availability

The datasets used and analysed during the current study are available from the corresponding author on reasonable request.
